# Population genomic data in spider mites point to a role for local adaptation in shaping range shifts

**DOI:** 10.1111/eva.13086

**Published:** 2020-08-27

**Authors:** Lei Chen, Jing‐Tao Sun, Peng‐Yu Jin, Ary A. Hoffmann, Xiao‐Li Bing, Dian‐Shu Zhao, Xiao‐Feng Xue, Xiao‐Yue Hong

**Affiliations:** ^1^ Department of Entomology Nanjing Agricultural University Nanjing China; ^2^ Bio21 Institute School of BioSciences The University of Melbourne Melbourne Victoria Australia

**Keywords:** invertebrate pests, local adaptation, range shifts, spider mites, whole‐genome resequencing

## Abstract

Local adaptation is particularly likely in invertebrate pests that typically have short generation times and large population sizes, but there are few studies on pest species investigating local adaptation and separating this process from contemporaneous and historical gene flow. Here, we use a population genomic approach to investigate evolutionary processes in the two most dominant spider mites in China, *Tetranychus truncatus* Ehara and *Tetranychus pueraricola* Ehara et Gotoh, which have wide distributions, short generation times, and large population sizes. We generated genome resequencing of 246 spider mites mostly from China, as well as Japan and Canada at a combined total depth of 3,133×. Based on demographic reconstruction, we found that both mite species likely originated from refugia in southwestern China and then spread to other regions, with the dominant *T. truncatus* spreading ~3,000 years later than *T. pueraricola*. Estimated changes in population sizes of the pests matched known periods of glaciation and reinforce the recent expansion of the dominant spider mites. *T. truncatus* showed a greater extent of local adaptation with more genes (76 vs. 17) associated with precipitation, including candidates involved in regulation of homeostasis of water and ions, signal transduction, and motor skills. In both species, many genes (135 in *T. truncatus* and 95 in *T. pueraricola*) also showed signatures of selection related to elevation, including G‐protein‐coupled receptors, cytochrome P450s, and ABC‐transporters. Our results point to historical expansion processes and climatic adaptation in these pests which could have contributed to their growing importance, particularly in the case of *T. truncatus*.

## 1 INTRODUCTION

Species range shifts are defined as any changes in the distribution of a species along latitude, longitude, and elevation or depth (marine ecosystems) over time (Lenoir & Svenning, 2015). The ranges of numerous species, as well as species composition and abundance in local communities, are shifting in response to climate change (Parmesan, 2006; Scheffers et al., 2016). A complex mosaic of local climate changes and associated biotic interactions involving temperature, precipitation, competitive release, and land‐use changes shape range shifts (Lenoir et al., 2010; VanDerWal et al., 2013). Changes in species abundance within a locality can represent intermediate states in an ongoing process of ranges shifting (Maggini et al., 2011). Species range shifts depend on inherent levels of species‐specific tolerance and the ability to which these can be modified by ongoing plastic changes and evolutionary adaptation (e.g., Atkinson, Siegel, Pakhomov, & Rothery, 2004; Jiguet et al., 2010; Moller, Rubolini, & Lehikoinen, 2008). Incorporating local adaptation in climate responses can greatly improve forecasts of species distribution and abundance under climate change (Bush et al., 2016; Peterson, Doak, & Morris, 2019).

Pest species particularly are expected to adapt to local climatic conditions given that short generation times and large population sizes in pests make rapid adaptation likely (Hoffmann, 2017). However, there is limited evidence for climatic adaptation in pests, with some notable exceptions being changes in diapause in planthoppers (Hou et al., 2016) and adaptive changes in thermal tolerance in earth mites (Hill, Chown, & Hoffmann, 2013). The importance of evolutionary factors in pest outbreaks is exemplified by adaptation to exogenous and endogenous toxins, such as pesticides (Kirk, Dorn, & Mazzi, 2013). On a longer evolutionary time scale, adaptation to host resources used by invertebrates has been linked to the expansion and contraction of gene families associated with detoxification (Rane et al., 2019).

While there is an increasing amount of information on the genomic basis of adaptation to pesticides and host plant toxins, there is much less information on adaptation in pests to other stresses. One reason why there is limited information on pest adaptation to climate is that the common garden and transplant experiments required to test for adaptation are rarely carried out, with only a few exceptions (Yadav, Stow, & Dudaniec, 2019). However, genomic data can also be used to obtain information on climatic adaptation (Rellstab, Gugerli, Eckert, Hancock, & Holderegger, 2015) particularly when a high density of markers is available across the genome, and samples are available across climatic gradients. Analyses of genomic variation are starting to be undertaken in pest species where invasion histories, range shifts, and demographic processes are understood and markers can then indicate signatures of selection. Knowledge of historical processes can help tracing the spread of invasive pests and the past conditions to which they have been exposed. This type of information can help in understanding and predicting potential pest invasion and outbreaks such as the likelihood of pest outbreaks occurring after invasion into new environments (Hoffmann, 2017).

Here, we undertake a detailed genomic study to investigate historical and current population processes affecting two agriculturally important and related spider mites, *Tetranychus truncatus* and *Tetranychus pueraricola* (Jin, Tian, Chen, & Hong, 2018). These spider mites have short generation times and large population sizes that should facilitate rapid adaptation across the range of climate conditions where they are found. The mites have genomes that are among the smallest in arthropods, making them suitable for whole‐genome resequencing (Grbic et al., 2011; Lasken, 2009). Both mite pests are common as well as being widespread: for instance, in a survey across the last ten years in mainland China, the occurrence frequency of *T. truncatus* on major crops reached 48.5%, followed by *T. pueraricola* at 21.4% (Jin et al., 2018), while in northern China, *T. truncatus* has become the main acarine pest of major crops including cotton, tomato beans and corn (Guo et al., 2013; Jin et al., 2018; Wang, Zhang, Wu, Xie, & Xu, 2013).

We report on whole‐genome sequencing of 173 *T* *. truncatus*, 67 *T* *. pueraricola,* and 6 individuals from sister groups. We investigated genomic diversity, population divergence, demographic history, and gene flow. We used these results to propose a scenario of how the spider mites originated and spread in China. We identified genes putatively associated with local adaptation to climate and elevation. We compared the number of genes putatively implicated in local adaptation for these two species with very different range sizes and abundances. The demographic and adaptation data in these two spider mites with different abundance were used to reveal important role that local adaptation may play in shaping range shifts.

## 2 MATERIALS AND METHODS

### 2.1 Sampling, amplification, and sequencing

We sampled large numbers of spider mites from fields in 2014–2017 across China. At each locality, we randomly collected up to three mites per plant, and plants were at least 1 m apart. We identified species through the nuclear ribosomal internal transcribed spacer (ITS) region (Ge, Ding, Zhang, & Hong, 2013). A total of 246 spider mites were selected for resequencing, including 173 *T* *. truncatus*, 67 *T* *. pueraricola,* and other *Tetranychus* species (Table S1).

Purified genomic DNA was extracted from female adult spider mites with DNeasy Blood & Tissue Kits following the manufacturer's protocol. In order to gain enough DNA for individual resequencing given the small size of mites (about 0.4 mm long; Fasulo & Denmark, 2000), whole‐genome amplification from purified genomic DNA was performed according to instructions of the QIAGEN REPLI‐g Ultrafast Mini WGA kit with some modifications. These included incubating the mixture of denaturation buffer and DNA at 95°C for 6 min and incubating the mixture of master mix and denatured DNA for multiple displacement amplification (MDA) at 30°C for 16 hr. Paired‐end 150 bp libraries with short inserts (~500 bp) were constructed according to the instructions of TruSeq Nano DNA Library Prep Kits. The whole‐genome sequencing was performed on an Illumina HiSeq 4,000 platform. Our target coverage was 10× per individual.

### 2.2 Alignment and SNP calling

Before mapping, raw reads were trimmed to obtain reliable clean reads using Trimmomatic 0.36 (Bolger, Lohse, & Usadel, 2014) under the following protocol: (a) cut adapter from the read; (b) perform a 4‐base wide sliding‐window trimming when the average quality per base drops below 15; (c) cut bases off both the start and the end of a read when below 5; and (d) drop reads below 50 bases long. The high‐quality reads were then aligned to the indexed *T. urticae* genome (Grbic et al., 2011) using bwa version 0.7.17 (Li & Durbin, 2009) with the mem command. The mapping results were transformed into BAM format and sorted using SAMtools v1.9 (Li et al., 2009). Instead of being removed, PCR duplicates were marked using the MarkDuplicates module of GATK version 4.0.12 (McKenna et al., 2010). We also used GATK4 to realign Indel regions of the indexed bam files and then identify variants across all individuals using the HaplotypeCaller module with default parameters. To reduce false variants, we applied hard filters to both SNPs and Indels in our raw variants based on the distribution of annotation values. For SNPs, we applied the following criteria: QD (variant confidence standardized by depth) <2.0; MQ (mapping quality of a SNP) <30.0; FS (strand bias in support for REF versus ALT allele calls) >60.0; and SOR (sequencing bias in which one DNA strand is favored over the other) >5.0. For Indels, we applied the following criteria: QD < 2.0; FS > 100.0; and SOR > 5.0. To get an analysis‐ready VCF file, we only kept SNP and Indel variants and biallelic sites.

### 2.3 Population structure

To infer evolutionary and genetic relationships of spider mites (e.g., *T. truncatus*, *T. pueraricola, T. urticae, T. kanzawai,* and *T. piercei*), we used a single VCF file with biallelic and high‐quality SNPs as an input into SNPhylo (Lee, Guo, Wang, Kim, & Paterson, 2014) to construct a maximum likelihood (ML) tree. Before determining a phylogenetic tree, we extracted SNP positions which met the criteria of minor allele frequency more than 0.01, missing rate less than 0.1 and *R* ^2^ < .6. Finally, we ended up with 16,251 SNPs for developing an ML tree with 1,000 bootstrap iterations and used *T. piercei* as the outgroup. To cluster spider mites by summarizing the major axis variation in allele frequencies, we conducted principal component analysis using the R package SNPRelate (Zheng et al., 2012) to obtain eigenvectors which could be plotted using the ggplot2 package (Wickham, 2016).

To infer ancestral proportions for each individual and suitable subgroups for populations of spider mites, ADMIXTURE (version 1.30; Alexander, Novembre, & Lange, 2009) analyses were performed with 10 replicate variant sets randomly down‐sampled to 10% of the high‐quality SNPs using different random seeds. Then, each replicate variant set was pruned to remove SNPs with minor allele frequency less than 1% and exclude SNPs above an *r* ^2^ threshold of 0.2 at sliding windows of 50 SNPs with a step size of 5 in PLINK v2.0 (Chang et al., 2015). Ten replicates of between 95,832 and 96,242 variants were used to carry out population structure analysis in ADMIXTURE 1.30 (Alexander et al., 2009). The number of assumed ancestral populations (*K*) ranged from 2 to 20 with fivefold cross‐validation and each *K* was repeated 10 times with different seeds based on the 10 replicate variant sets, resulting in a total of 100 runs for each value of *K*. Then, we used CLUMPAK (Kopelman, Mayzel, Jakobsson, Rosenberg, & Mayrose, 2015) with default settings to provide a better understanding of different solutions reported by ADMIXTURE. CLUMPAK generated average results of Q‐matrices for each value of *K* from all runs, so that we could plot ancestry proportions for all individuals.

To infer population‐level splits and mixtures for both *T. truncatus* and *T. pueraricola* populations, we filtered the high‐quality SNPs obtained by GATK with the following criteria: the max missing rate was 0.9, and *r* ^2^ should be less than 0.2. Then, we used Treemix 1.13 (Pickrell & Pritchard, 2012) to investigate the admixture between populations, with migration edges ranging from 1 to 20 and *T. piercei* as outgroup.

### 2.4 Analyses of genetic diversity, ROH, and LD

To reduce the influence of statistical bias caused by low coverage and unidentical sample sizes of different populations, we calculated population differentiation (*F*_ST_) and nucleotide diversity (*θ*) in ANGSD 0.929 (Korneliussen, Albrechtsen, & Nielsen, 2014). For the SFS computation, we implemented the following criteria: (a) only keep uniquely mapped reads; (b) use only sites with data from at least 80% individuals; (c) ensure the minimum total depth equals the sample sizes of the group; and (d) ensure both the minimum mapping quality and base quality equal 30. The ancestral state was assigned with the outgroup *T. piercei* to polarize allele state. Then, realSFS and thetaStat programs were used to estimate *F*_ST_ and *θ*. The regions of homozygosity (ROH) for each population were calculated in the program BCFtools 1.9 (Narasimhan et al., 2016). Linkage disequilibrium (LD) decay analysis for each population/group was measured in PopLDdecay (Zhang, Dong, Xu, He, & Yang, 2018) with the following parameters: ‐MaxDist (maximum distance (kb) between two SNP) 200 and –MAF (minimum minor allele frequency filter) 0.01.

### 2.5 Demographic history

We used pairwise sequentially Markovian coalescent (PSMC) models to estimate variation in effective population size (*N*_e_) of different species of *Tetranychus* (*T. urticae* from Canada; *T. kanzawai, T. truncatus,* and *T. pueraricola* from China) based on density of heterozygotes (Li & Durbin, 2011). The parameters were set as follows: ‐N25 ‐t15 ‐r5 ‐p “4 + 25*2 + 4+6”. The description of each parameter and scripts is available on https://github.com/lh3/psmc. For *Tetranychus*, we assumed a mutation rate of 1 × 10^−9^ mutations per base pair per generation and a generation time of 1 month, modifying parameters from *Drosophila* (Keightley, Ness, Halligan, & Haddrill, 2014). The historical effect of recent effective population size was estimated with SMC++ which requires only unphased genomes and provides more accurate estimates for the recent past taking advantage of linkage disequilibrium information in coalescent hidden Markov models (Terhorst, Kamm, & Song, 2017). The default settings resulted in too much oscillation as well as overfitting of the curves for estimating recent effective population size of spider mites. To correct this issue, we experimented with various parameters suggested by SMC++ documentation to identify the settings best suited for our data. Finally, we specified polarization error of 0.5 as the identity of the ancestral allele is not known, and a regularization penalty of 0.5 to shrink too much oscillation of the estimated size history. To prevent overfitting, we specified a threshold of 1e‐2 for stopping the EM algorithm when the relative improvement in log‐likelihood became small. The estimation command was “‐‐cores 25 ‐‐timepoints 20 2e6 ‐knots 3 ‐‐thinning 600 ‐‐spline cubic ‐‐ftol 1e‐2 ‐‐polarization‐error 0.5 ‐rp 0.5 1e‐9”.

Inferences about the demographic history were made through a continuous‐time coalescent simulator fastsimocal2 (Excoffier, Dupanloup, Huerta‐Sánchez, Sousa, & Foll, 2013) which estimates parameters from a site frequency spectrum (SFS). To reduce the biases in SFS caused by sequencing error and coverage depth, we computed SFS based on the realigned bam files output from GATK4 using ANGSD 0.929 (Korneliussen et al., 2014). To avoid any kind of selection influence on demographic inference, we used only intergenic SNPs. After generating allele frequency likelihood files under GATK genotype likelihood model, we used realSFS program in ANGSD (Korneliussen et al., 2014) to estimate joint SFS. The joint SFS across different groups was used to estimate demographic parameters under 35 alternative models of phylogenetic relationships and historical events (Table S8). For each model, we ran fastsimcoal2 with 100,000 simulations to estimate the expected derived SFS, 40 conditional maximization (ECM) cycles and 50 times for estimating the parameters. SFS entries with less than 10 SNPs were pooled together. The best‐fitting model was assessed through the likelihood and Akaike information criterion (Excoffier et al., 2013). Parametric bootstrap estimates were obtained with 50 data sets simulated with estimates of parameters of the best model.

### 2.6 Local adaptation analysis

To detect genomic signatures of adaptation to the local climate and elevation, we performed a gene‐environment association study using the whole‐genome resequencing data. The climate and elevation data set (Table S10) were downloaded from CHELSA (Karger et al., 2017) and from USGS (Danielson & Gesch, 2011). We summarized the climatic variables by keeping half of the positive PCNMs (principal coordinates of neighbor matrices) for temperature variables and for precipitation variables. To get a genotype matrix, we used VCFtools v0.1.13 (Danecek et al., 2011) to preprocess SNPs by removing sites with MAF < 0.05, missing rate >0.1 and individuals with relatedness >0.0884. Then, the missing values of genotype were imputed with the program Beagle 5.0 (Browning, Zhou, & Browning, 2018) to improve the accuracy of the genotypes. After all these filtering steps, we had 120 individuals with 262,541 SNPs for *T. truncatus* and 51 individuals with 401,999 SNPs for *T. pueraricola*. To control the false discovery rate (FDR), we, respectively, chose appropriate value ranges of *K* = 12–16 and *K* = 4–7 for latent factors of *T. truncatus* and *T. pueraricola* based on the ADMIXTURE analysis (Figure S4). Then, we ran 5 repetitions of the latent factor mixed model to compute new calibrated *P*‐values in LFMM v1.5 (Frichot, Schoville, Bouchard, & François, 2013). The lists of candidate loci were obtained after controlling FDR at the level *q* = 0.5% using the Benjamini–Hochberg procedure. As low dispersal ability of spider mites might increase false‐positive rates with LFMM (Forester, Jones, Joost, Landguth, & Lasky, 2016), we also used redundancy analysis (RDA) to identify signatures of local adaptation following the methods and scripts referred in Capblancq, Luu, Blum, & Bazin (2018). Briefly, individual genotypes and PCNMs for climatic variables obtained from the above LFMM were used as the response matrix and explanatory matrix, respectively. The parameters used in the RDA analysis were the same as in Capblancq et al. (Capblancq et al., 2018) except for controlling FDR at the level *q* = 0.5% to identify outlier loci involved in local adaptation.

We reconstructed PCA for all candidate SNPs associated with local adaptation identified by LFMM to regroup populations of spider mites following the same PCA procedure described above for analyzing population structure. We used a sliding‐window approach (100‐kb windows sliding in 10‐kb steps) to calculate the genome‐wide distribution of genetic differentiation (*F*_ST_), pairwise nucleotide variation (*θ*_π_) ratios and selection statistics (Tajima's *D*) between different groups of the two species. These values were calculated with VCFtools v0.1.13 (Danecek et al., 2011) to detect selection signals for candidate loci.

## 3 RESULTS

### 3.1 Sequencing and variation

Our large‐scale sampling of spider mites from 39 locations in 2014–2017 mainly in mainland China (Figure S1) identified *T. truncatus* and *T. pueraricola* as the two most dominant species with different range shifts on crops, which agrees with a previous long‐term survey (Jin et al., 2018). After whole‐genome amplification and resequencing, we generated genomes of 173 *T* *. truncatus* from 17 populations, 67 *T* *. pueraricola* from 10 populations, and 6 individuals of outgroups (*T. urticae, T. piercei, T. kanzawai,* and *T. parakanzawai;* Figure S1 and Table S1). Most of our samples were collected from China, except for 6 *T* *. truncatus* from Japan and 2 *T* *. urticae* from Canada (Table S1). We generated a total of 285 Gb aligned high‐quality bases at a total of 3,133 × effective depth and an average of 13 × depth per individual (Table S2).

After filtering, we obtained 4.46 million SNPs for *T. truncatus* and 3.74 million SNPs for *T. pueraricola* with 0.95 million SNPs shared in these two species (Table S3). For *T. truncatus*, 3.58 million SNPs (80.3%) were located in non‐coding regions and 0.88 million SNPs (19.7%) were located in coding regions: 10.6% were synonymous and 7.5% were non‐synonymous, with a non‐synonymous/synonymous ratio of 0.707. For *T. pueraricola*, 3.02 million SNPs (80.7%) were located in non‐coding regions and 0.72 million SNPs (19.3%) were located in coding regions: 9.6% were synonymous and 7.8% were non‐synonymous, with a non‐synonymous/synonymous ratio of 0.815 (Table S4). To scale the distribution of SNPs across the *T. urticae* reference for all these 6 species, we calculated density of SNPs in bins of 100kb size and found that SNPs of all 6 spider mite species were mainly located in the first 50 scaffolds, while those of *T. pueraricola* were more commonly distributed in the rest of the scaffolds (Figure S2).

### 3.2 Population structure of the most two dominant spider mites

**FIGURE 1:**
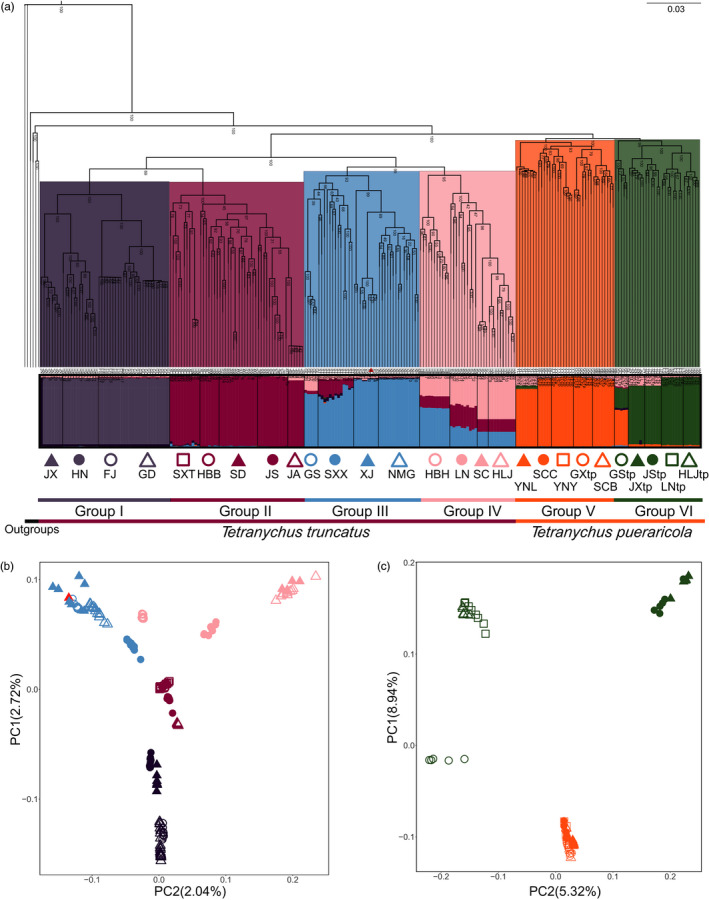
Population genetics analyses of *T. truncatus* and *T. pueraricola*. (a) Phylogenetic tree (maximum likelihood) and the ADMIXTURE analysis (*K* = 6) inferred from whole‐genome SNPs of spider mites, with *T. piercei, T. kanzawai, T. parakanzawai,* and *T. urticae* as outgroups. In ADMIXTURE analysis, each horizontal bar represents an individual. All populations are represented by open and filled symbols which are used in the PCA in the next panel. (b) Principal components analysis (PCA) of *T. truncatus* and (c) *T. pueraricola*. An outlying individual of the SC population denoted as a solid triangle may reflect a sampling error

The phylogenetic and ADMIXTURE analyses clearly separated the two species (*T. truncatus* and *T. pueraricola*; Figure 1a). A few individuals exhibited ancestral proportions from both species; one JXtp individual of *T. pueraricola* had the largest ancestral proportion of *T. truncatus* when *K* = 2 (7%; Figure S3). The results of cross‐validation error suggested that the best *K* was 15, which makes it complicated to classify mite populations (Figure S3). The ancestral proportions of separate ADMIXTURE analyses (Figure S4) were consistent with the combined analyses (Figure S3), with the best *K* of 12 and 5 for *T. truncatus* and *T. pueraricola*, respectively. As 4 paraphyletic groups of *T. truncatus* and the 2 paraphyletic groups of *T. pueraricola* are suggested by phylogenetic tree (Figure 1a), we used the *K* = 6 ADMIXTURE result to cluster all populations of the spider mites in China into six main groups that were geographically associated. We used these six clusters in demographic analyses as well. Group I – Group IV comprised *T. truncatus* populations from southern China (provinces Jiangxi, Hunan, Fujian, and Guangdong; JX, HN, FJ, and GD), eastern China (provinces Shanxi, Hebei, Shandong, Jiangsu; SXT, HBB, SD, and JS) and Japan (JA), midland and northwestern China(provinces Gansu, Shaanxi, Xinjiang, and Inner Mongolia; GS, SXX, XJ, and NMG), northeastern China (provinces Hebei, Liaoning, Sichuan, and Heilongjiang; HBH, LN, SC, and HLJ); Group V and Group VI comprised *T. pueraricola* populations from southwestern China (provinces Yunnan, Sichuan, and Guangxi; YNL, SCC, YNY, GXtp, and SCB) and other areas of China (provinces Gansu, Jiangxi, Jiangsu, Liaoning, and Heilongjiang; GStp, JXtp, JStp, LNtp, and HLJtp).

**FIGURE 2:**
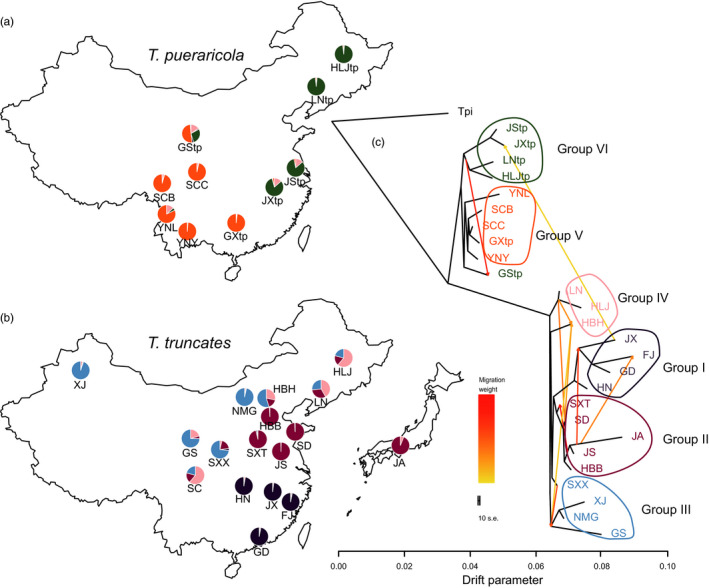
Maps and admixture events of the spider mites. Geographic locations of 27 populations for *T. pueraricola* (a) and *T. truncatus* (b) with ancestral proportions (*K* = 6) inferred from ADMIXTURE. (c) The relationships among populations of these two dominated spider mites. Each population is colored according to its corresponding group, and each arrow indicates a migration event which is colored with migration weights. The scale bar shows 10× the average standard error of the entries in the sample covariance matrix

The principal component analysis (PCA) supported the grouping results (Figure 1b,c). The first axis of the PCA explained a high degree of variance for the overall data set including *T. truncatus* and *T. pueraricola* (41.32%, Figure S5a) indicating the clear differences between the species. Both the PCA of *T. truncatus* and *T. pueraricola* mirrored the geography of the sampled populations (Figure 2a,b), which highlighted historical migrations or reflected “isolation by distance” (Reich, Price, & Patterson, 2008). PC1 of *T. truncatus* aligned along the south/north direction of China and accounted for 2.72% of the variation. PC1 of *T. pueraricola* aligned in a southwest/northeast direction and accounted for approximately three times as much of variation as the first axis in *T. truncatus* (8.94% vs. 2.72%).

The ML tree inferred from Treemix explained up to 99.99% of the variance between populations when being added with 10 migration edges (Figure 2c). Among 10 migration events, half were related to Group II, including within Group II, from Group II to Group I, and from Group II to Group IV. The lower genetic differentiation (*F*_ST_) between Group II and other groups (Table S7) also supported higher gene flow. There was introgression between *T. truncatus* and *T. pueraricola* in Jiangxi (JX and JXtp).

### 3.3 Nucleotide diversity, population differentiation, and linkage disequilibrium

The average nucleotide diversity (*θ_π_*) for the 4 spider mites (*T. truncatus, T. pueraricola, T. piercei,* and *T. urticae*) was 5.54 × 10^−4^. The genome‐wide *θ_π_* value ranged from 2.74 × 10^−4^ to 6.66 × 10^−4^ and 3.59 × 10^−4^ to 1.06 × 10^−3^ for *T. truncatus* and *T. pueraricola,* respectively, (Table S5). Despite a wider geographic distribution and a higher abundance (Jin et al., 2018), *T. truncatus* had lower genetic diversity than *T. pueraricola* as a whole (Figure S6a,b). The pattern of genetic diversity remained the same in species comparisons from the same locations where both species were sampled (GS, JX, JS, LN, and HLJ).

The *F*_ST_ values among different populations of the two spider mites varied markedly (*T. truncatus*, 0.041‐0.809; *T. pueraricola*, 0.115‐0.748; Table S7). The *F_ST_* values between remote populations were higher than between adjacent populations, suggesting the absence of long‐distance migration in most instances. The regions of homozygosity (ROH) were fewer and shorter for both *T. truncatus* and *T. pueraricola* in average number and size in southern (Group I) and southwestern (Group V) China (Table S6 and Figure S6c–f), indicative of larger populations or admixture (Ceballos, Joshi, Clark, Ramsay, & Wilson, 2018). The range of LD decay distances of *T. truncatus* populations was wider than that of *T. pueraricola* populations due to one outlier population, but otherwise, the two species showed similar levels of decay (Figure S7). These genetic parameters suggest distinct evolutionary and demographic histories for some *T. truncatus* and *T. pueraricola* populations.

### 3.4 Demographic history

**FIGURE 3:**
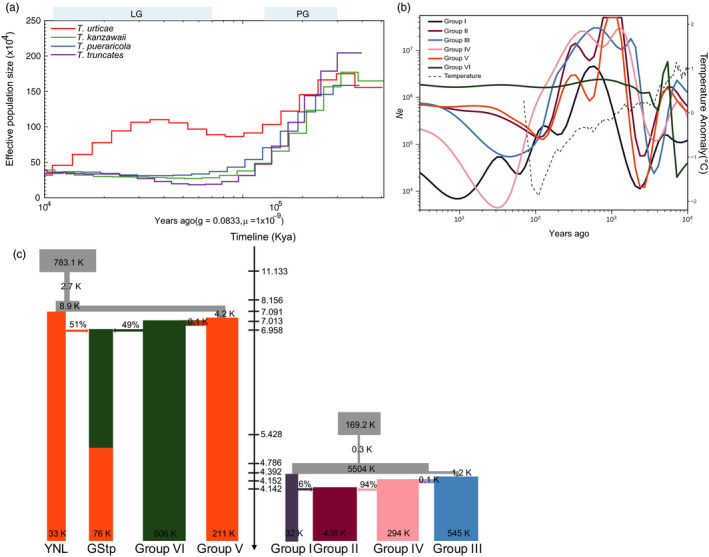
Demographic history of the spider mites. (a) PSMC analysis performed on the representatives of each species sequenced at high coverage to indicate variation in *N*_e_ over the last 10^5^ years. LG and PG represent period of last glaciation and penultimate glaciation separately. (b) Demographic changes of *N*_e_ for six groups of *T. truncatus* and *T. pueraricola* in the recent 10^4^ years using SMC++. Note that the SC population has been excluded from Group IV because of sampling uncertainty. Northern hemispheric (90‐30N) temperature anomaly from the mean temperature of AD 1962–1990. (c) The best demographic scenarios for *T. truncatus* and *T. pueraricola* inferred by fastsimcoal2. The gray rectangles represent ancestral populations, and the arrows indicate admixture events. To better understand demographic history of *T. pueraricola*, populations of YNL and GStp are assigned as single groups

With single representatives of each species sequenced at higher coverage, we estimated historical changes in the effective population size (*N*_e_) by pairwise sequentially Markovian coalescent analysis (Li & Durbin, 2011). The historical *N*_e_ of *T. urticae* sampled in Canada exhibited different demographic patterns to other Asian spider mites (Figure 3a). The ancestors of all spider mites reached their peak *N*_e_ at about 0.21 Mya (million years ago; Figure 3a) during interglacial periods before the Penultimate glaciation. Then, in the period of Penultimate glaciation, both North American and Asian populations experienced bottleneck events which were more dramatic in Asian populations. At about 80 Kya (thousand years ago), the *N*_e_ of North American and Asian populations reached their lowest point and then began to increase. Since the last glaciation 40 Kya, the North American population has been declining, whereas even across the LGM (Last Glacial Maximum, 26.5 Kya BP), Asian populations have been gradually expanding (Figure 3a).

As PSMC analysis has limited resolution in the recent past, we implemented SMC++ (Terhorst et al., 2017) to infer recent *N*_e_ history of the 6 groups of *T. truncatus* and *T. pueraricola*. The default settings in SMC++ did not suit our species well, producing oscillation in *N*_e_. By experimenting with various parameters, we obtained a clearer recent historical pattern of *N*_e_ which partly mirrored fluctuations in northern hemispheric temperature (Figure 3b; Marcott, Shakun, Clark, & Mix, 2013). This pattern suggests that each group experienced bottleneck events during 4–2 Kya and then increased to at least 10^6^_,_ followed by a sharp decrease over hundreds of years.

Based on the population structure analysis (Figure 1), we assigned YNL and GStp populations as single groups to infer demographic history. Among a total of 35 alternative models of demographic diffusion detected by fastsimcoal2 (Excoffier et al., 2013), we found that model 15 for *T. truncatus* and model 14 for *T. pueraricola* achieved the maximum log‐likelihood values and these were selected as the optimal models (Table S8). According to these models, the migratory directions of *T. truncatus* and *T. pueraricola*, from south to north and southwest to northeast, were consistent with the first PCs (Figure 1c,d). The *T. pueraricola* populations from eastern and northern China were predicted to have diverged from the southwestern lineage, estimated as ~7,031 years ago, soon after the initial divergence among the original southwestern populations, estimated as ~7,091 years ago. There was a comparably higher effective population size (*N*_e_ = 506K) in colonized regions. Meanwhile, a hybrid population, GStp, in midland China was derived from admixture of the ancestors of YNL (43%) and Group VI (57%) in a short time (Figure 3c). The *T. truncatus* populations from midland and northern (Group III) China diverged ~4,392 years ago from the lineage ancestral to contemporary southern populations, with an effective population size of 545K. Then, the southern lineage admixed with northern populations which diverged an estimated ~4,152 years ago from Group III eastern populations (Group II) and emerged as the eastern lineage Group II.

### 3.5 Genomic signatures of local adaptation

**FIGURE 4:**
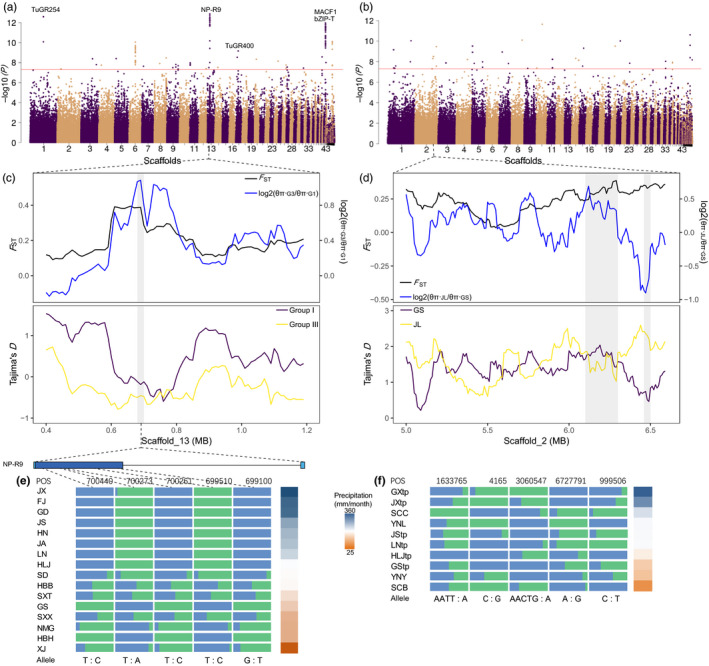
Signatures of local adaptation in the genome of spider mites. Manhattan plots of adjusted *p*‐values using the LFMM for association between SNPs and precipitation in *T. truncatus* (a) and *T. pueraricola* (b). The genome‐wide significance threshold (‐log10(5e‐8)) is indicated by the red horizontal line. Those genes with non‐synonymous substitution caused by significantly associated SNPs (adjusted *p*‐value > 5e‐8) were marked in the Manhattan plots. (c) *F_ST_*, log_2_(*θ*_π_ ratio), and Tajima's *D* values around the strongest associated gene NP‐R9 of *T. truncatus*. G1 and G3 are the abbreviations for Group I and Group III. (d) *F_ST_*, log_2_(*θ*_π_ ratio), and Tajima's *D* values around significantly associated genomic regions in scaffold_2 of *T. pueraricola*. JL represents JStp and LNtp populations. GS represents GXtp and SCB populations. (e) Allele frequencies of five non‐synonymous mutations within the NP‐R9 gene across the populations of *T. truncatus*. Based on the precipitation of the wettest month, the relatively moist regions include FJ, GD, JX, and HN and the relative arid regions include XJ, NMG, GS, and HBH. (f) Allele frequencies of the five most associated SNPs with the genomic positions of scaffold_10:1633765, scaffold_230:4165, scaffold_8:3060547, scaffold_1:6727791, and scaffold_21:999506 across the populations of *T. pueraricola*

To investigate genetic basis of local adaptation, we identified genomic variants associated with temperature, precipitation, and elevation using latent factor mixed models which accounted for background structure (Frichot et al., 2013). After several runs, we chose *K* = 16 and *K* = 7, respectively, for *T. truncatus* and *T. pueraricola* for which the estimates of genomic inflation factors (λ) were close to 1.0 (Figure S8). However, the unsmooth histograms of *p*‐values (Figure S8) indicated that confounding effects in *T. pueraricola* could not be well controlled. The *p*‐value histograms of all variables for *T. truncatus* appear flat except for a peak close to 1 which may reflect genotypes at these sites differing in only one or two populations. In *T. truncatus*, we found 329, 705, and 1,493 SNPs associated with temperature, precipitation, and elevation, respectively (expected FDR = 0.5%; Figure 4, Figure S9). In *T. pueraricola,* the equivalent figures were 2, 208 and 1,241 SNPs, respectively. Most of the SNPs associated with local adaptation located in the upstream and downstream regions (5kb) followed by the exon region (Table S11). The RDA analysis also identified more outlier loci involved in local adaptation for *T. truncatus*, including 72% of the candidate loci detected by LFMM, than for *T. pueraricola* (4,090 vs. 0 SNPs; Figure S10). As LFMM is more powerful for detecting outliers under weak selection (Forester et al., 2016), we paid more attention to the candidate loci detected by LFMM. The functional variants caused amino acid substitutions in 51, 76, and 135 genes in *T. truncatus* associated with temperature, precipitation, and elevation, respectively, and 0, 17, and 95 genes in *T. pueraricola* associated with these factors, respectively (Tables S12–S14). Amino acid substitutions affect the activity, folding, or assembly of the protein and may have measurable effects on fitness. These type of changes may contribute to local adaptation in this study.

The LFMM analysis indicated a number of SNPs that were significantly associated with precipitation (adjusted *p*‐value ≤ 5e‐8) in *T. truncatus*, higher than for *T. pueraricola* (Figure 4a,b). There were also several contiguous regions with adaptive loci detected in *T. truncatus*, but not in *T. pueraricola*. The PCA based on candidate SNPs associated with precipitation showed that *T. truncatus* was clearly clustered to moist (Group I, precipitation of wettest month > 220 mm/month) and arid (Group III, precipitation of wettest month < 130 mm/month) regions, while clusters for *T. pueraricola* were not related to precipitation (Figure S11 and Table S10). The first PC of *T. truncatus* accounted for almost fivefold as much of the variance as the second PC (33.77% versus. 7.02%) and that of *T. pueraricola* was 1.7 × higher (25.13% vs. 15.03%), which suggested that precipitation may play a more important role in local adaptation by *T. truncatus*.

We estimated population genetics parameters of the extreme populations (Group I and Group III of *T. truncatus*; JStp, LNtp, and GXtp, SCB populations of *T. pueraricola*) based on the PCA of these two spider mites (Figure S11 and S12). The strongest association (adjusted *p*‐value = 1.3e‐13; Figure 4a) between genotype and precipitation in *T. truncatus* was adjacent to the NP‐R9 gene (tetur13g01650). The target gene NP‐R9 exhibited markedly higher *F*_ST_and log_2_(*θ*_π_ ratio) but lower Tajima's *D* values (Figure 4c) than adjacent genomic regions, suggesting a selective sweep. Within NP‐R9, allele frequencies of several non‐synonymous substitutions varied with precipitation (Figure 4e), suggesting an important role for this gene in local adaptation. However, the associated loci of *T. pueraricola* detected by LFMM exhibited little evidence for population genetic signatures of natural selection. For example, the *F*_ST_, log_2_(*θ*_π_ ratio), and Tajima's *D* values of associated loci were not always significantly different from adjacent regions (Figure 4d). The allele frequencies of the top 5 hit SNPs showed unclear patterns of distribution among the populations of *T. pueraricola* (Figure 4f).

For elevation, large numbers of SNPs showed associations in both spider mites (Figure S9). The reconstructed PCA of elevation‐associated SNPs clustered with the elevation gradient alongside PC1 for both species (Figure S12, Table S10), with PC1 for *T. pueraricola* accounting for a similar amount of variation as for *T. truncatus* (34.83% vs. 27.73%). The genome areas associated mostly with significant loci were upstream of the neuropeptide receptor NP‐R9 (Figure S9c) which could play a central role in evolutionary adaptation of *T. truncatus*. For *T. pueraricola*, the most significantly associated SNPs resulting in amino acid replacements were inconsistent with *T. truncatus* and had potential functional effects on gustatory receptors, X‐prolyl aminopeptidase, immunoglobulin, and laminin.

## 4 DISCUSSION

Our genomic study provides information on the evolutionary histories of the two dominant mites in China including the possible origin of the pests, their range shifts, demographic histories, and potential genes involved in local adaptation. Although having lower genetic diversity and spreading to colonize new areas ~3,000 years later than *T. pueraricola*, *T. truncatus* has become more prevalent and more abundant after expanding its range to arid northern China. *T. pueraricola* remains common in areas close to its origin rather than in expanded ranges.

A species difference in the degree of local adaptation may have shaped the extent of range shifts in these spider mites and changes in species abundance. Our comparative genome‐environment association analyses uncovered significantly different *F*_ST_, log_2_(*θ*_π_ ratio), and Tajima's *D* values of candidate loci in *T. truncatus* along with more genes strongly associated with precipitation, while adaptation to precipitation in *T. pueraricola* appeared weaker. These results suggest that adaptation to climate might also influence the relative ranges of these pests in the future. Understanding how pests respond to climate stresses can aid in predicting future invasion and outbreaks. For instance, an invasive distribution range may be under‐predicted when temperature adaptation occurs, as in the case of *Halotydeus* and *T. evansi* mites (Hill et al., 2013; Migeon et al., 2009). Local adaptation may result in spider mites being pests in areas where they have previously not been observed, resulting in the need for additional control measures in those areas.

### 4.1 Linking demographic and evolutionary histories to range shifts of spider mites

The favored models of population expansion (Figure 3c) suggested that both *T. truncatus* and *T. pueraricola* in China originated from southern and southwestern China. Southwestern China and northern Vietnam have provided long‐term stable refugia for many relict plants (Tang et al., 2018), and have been proposed as possible origin regions for some species such as pears and tigers (Liu et al., 2018; Wu et al., 2018). Despite its absence in southwest China, *T. truncatus* may also originate from refugia near southwestern China because of identical fluctuation of ancestral effective population size during LGM (Figure 3a). The long‐term climatically stable refugia probably provided host plants and suitable environments for protecting ancient populations of Asian spider mites. The species richness of spider mites in southern China is also higher than that in northern China (Jin et al., 2018), which likely reflects the fact that the southwestern area provided refugia for many invertebrates. Although there are parallel patterns of expansion between the mite species, the early expansion of *T. urticae* is intriguing and may indicate a higher tolerance of conditions existing during glaciation periods or movement of this species from other areas that aided population expansion.

We had expected the value of best *K* in the ADMIXTURE analysis to be 2 (Figure S3), reflecting two distinct species of *Tetranychus*. The resulting best K as 15 for 27 populations of *T. truncatus* and *T. pueraricola* reflects the substantial genetic differentiation between populations within species, while with 2 subgroups assumed there are departures from Hardy–Weinberg equilibrium. The favored models (Figure 3c) suggested migration directions of south to north and southwest to northeast in China for *T. truncatus* and *T. pueraricola,* respectively. The importance of these directions in the demographic history of these spider mites was supported by the PC1 axis of PCA (Figure 1b,c). Some population expansion patterns supported by the favored models were also supported by Treemix, for example, migration event from ancestors of Group VI to GStp (Figure 2c). However, the paraphyletic topology of the mite species suggested by the ML tree (Figure 1a) demonstrated unclear evolutionary relationships between the groups, probably arising from long‐term isolation. Intriguingly, we note that *T. truncatus* in Japan probably originated from locations near Jiangsu in China, from which small brown planthoppers in Japan are also thought to have originated (Otuka et al., 2010). The association between Jiangsu and Japan could be further tested by characterizing additional Japanese populations. In addition, we note a close genetic relationship between geographically distant populations of *T. truncatus* (SC and HLJ; Figure 1a and Table S7). These close affinities likely represent the result of human‐mediated movement such as through trade.

After experiencing a dramatic decrease, *N*_e_ of *T. truncatus* in northern and midland China (Group II, Group III, and Group IV) began to increase almost 30 years ago (Figure 3b), which is consistent with historical reports. Since the mid‐1980s, with the expansion of wheat, corn, and soybean plantings, combined with warm and drought conditions, *T. truncatus* has increased from being a minor pest to a major pest (Hong, 2012). Populations of *T. truncatus* from Group I had the lowest level of genetic diversity (Figure S6a) and the greatest estimated fluctuations in *N*_e_ based on a coalescence analysis of LD patterns (Figure 3b). Note that this Group also showed the highest positive Tajima's *D* relative to other groups (Figure S13) which indicates population contraction.

The average genome‐wide *θ_π_* (5.54 × 10^−4^) of spider mites was lower than estimates for most animals and plants that have been characterized, such as the giant panda (1.24 × 10^−3^; Zhao et al., 2013), *Anopheles* mosquito (1.5 × 10^−2^; Consortium, 2017), and cultivated rice (5.4 × 10^−3^; Xu et al., 2011). The relatively lower genetic diversity of *T. truncatus* may represent recent differences in population processes in this species compared to *T. pueraricola*. Range‐limit theory proposes that abiotic factors form high‐latitude/altitude limits, whereas biotic interactions create lower limits (Siren & Morelli, 2020). Perhaps the high standing genetic diversity of *T. pueraricola* relates to biotic factors such as competition, predation, and pathogens producing heterogeneous environments which has been hypothesized for a long time as facilitating the maintenance of genetic variation (McDonald & Ayala, 1974), and contribute to the widespread distribution of this species in low latitude areas. A relatively low genetic diversity does not appear to have impeded the spread of *T. truncatus* to high‐latitude areas where conditions are more homogeneous and directional selection associated with abiotic factors (e.g., cold climate) is expected to be intense. Given that high genomic diversity can facilitate adaptive shifts, *T. pueraricola* in Gansu with high diversity (Figure S6a) may adapt to future conditions and lead to a range expansion of this acarid pest, particularly if rare alleles are involved in the adaptive shifts.

According to field surveys (Guo et al., 2013; Jin et al., 2018; Wang et al., 2013), *T. truncatus* was the most abundant and widespread acarid pest in areas of Group II, Group III, and Group IV; *T. pueraricola* was the most prevalent in areas of Group V. The different patterns of range shifts of these two spider mites may be linked to several factors. First, monoculture farming in northern China increased ecological homogeneity, leading to the possibility of *T. truncatus* to spread. Low levels of biodiversity caused by agricultural intensification generally benefit agricultural pests by reducing natural enemies and supplying abundant resources (Benton, Vickery, & Wilson, 2003; Hong, 2012; Sotherton & Self, 2000). A simplified landscape may allow for the rapid growth of pest populations, which can facilitate rapid adaptation (Reznick & Ghalambor, 2001). Second, *T. truncatus* in its prevalent regions (Group II, Group III, and Group IV) had higher gene flow (Figure 2c), which could have contributed to a higher level of genetic variability (Table S5) and more rapid range shifts of those regions. Third, results here suggested that *T. truncatus* were locally adapted to climatic conditions which could benefit range shifts. Although many factors interact with climate change to alter range shifts, the potential role of local adaptation has been highlighted theoretically in driving nonintuitive patterns of range shifts (Louthan, Doak, & Angert, 2015; Suttle, Thomsen, & Power, 2007). Strong local adaptation may lead an expanded range of climate tolerances for a species as a whole. In southern China which likely represents the region where *T. truncatus* originated, a relatively low *N*_e_ (Figure 3c) may be the result of weak adaptation to the hot‐humid environment and limited gene flow or others factors such as competition with other spider mites. In contrast, local adaptation together with high gene flow of *T. truncatus* in northern China may contribute to relatively high performance under arid conditions. The genetic parameters of genome regions around candidate loci also suggest adaptation to dry conditions in *T. truncatus* but less clear cut signals of adaptation for *T. pueraricola*. However, these remain conjectures and need to be tested. In particular, quantitative genetic experiments are required to assess the heritability of traits linked to tolerating different climate conditions. And common garden experiments could indicate the extent of local adaptation across the species to see whether these match expectations based on the genomic data.

### 4.2 Genes involved in local adaptation

Many SNPs were significantly associated with climate and elevation in *T. truncatus* and *T. pueraricola*, and we focused on the genes with amino acid substitutions. There were less SNPs genome‐wide significantly associated (adjusted *p*‐value ≤ 5e‐8) with temperature than with precipitation and elevation in both mites (Figure S9). Along with the recent historical *N*_e_ which partly reflected past temperature, these may indicate spider mites are susceptible to temperature selection. There has been much research on temperature effects on development, fecundity, longevity, and pesticide impacts in spider mites (Auger, Guichou, & Kreiter, 2003; Margolies & Wrensch, 1996; Riahi, Shishehbor, Nemati, & Saeidi, 2013). Under intensifying global warming, spider mites will increasingly threaten agricultural production (Migeon et al., 2009). Our results provide a framework for genome‐wide association studies to locate key genes that may be responsible for further adaptation to temperature changes in spider mites.

Many genes were found to be significantly associated with precipitation in *T. truncatus*. Three genes with amino acid replacements, NP‐R9, TuGR254, and TuGR400 (Figure 4a), belong to members of the G‐protein‐coupled receptor (GPCR) family. When interacting with insect neurohormones, these genes could play a role in the control of development, behavior, feeding, reproduction, and many other physiological processes (Hauser et al., 2008; Martin, Boto, Gomez‐Diaz, & Alcorta, 2013). A vasopressin‐like GPCR found in *Tribolium* might help this xerophilous insect to effectively control water reabsorption (Hauser et al., 2008). Variation in these GPCRs might contribute to the regulation of homeostasis of water and ions (Gäde, 2004), and thereby be involved in adaptation to the dry environment in northern China. Other significantly associated genes including tetur43g00160 and tetur43g00520 were annotated to bZIP‐T and MACF1 (Figure 4a). The bZIP‐T gene encodes a basic leucine zipper‐type protein which participate in an abscisic acid‐dependent signal transduction pathway when *Arabidopsis* face drought and high‐salinity environments (Uno et al., 2000). The reduction of MACF1 caused by heterozygous duplication resulted in periodic hypotonia, lax muscles and diminished motor skills in human (Jørgensen et al., 2014). The NP‐R9 and its upstream are involved in adapting to precipitation and elevation, and may serve as target regions for further functional experiment. However, these genes need to be further characterized through functional studies.

Although both *T. truncatus* and *T. pueraricola* showed many genes associated with elevation, many of them are not well annotated (Table S14). A dense region of significantly associated SNPs on scaffold_8 (Figure S9c) included amino acid replacement in 4 genes which encoded a hypothetical protein, Agrin, and Myc‐type transcription factors. A cytochrome P450 gene, CYP389C5, had a non‐synonymous substitution, C100840T, which only appeared in high‐elevation populations (GS, SXX, NMG, and HBH). Cytochrome P450 enzymes have been related to high‐elevation adaptation in Tibetan human populations (Simonson et al., 2010). Other strongly associated genes that had amino acid replacements, such as TuABCH‐21 (ABC‐transporter) and HR96‐like h (HR96‐like nuclear receptor h), have not been previously linked to high‐elevation conditions and need further study. The results here suggest that the related mite species have different strategies when adapting to high elevations.

The higher number of SNPs associated with local adaptation and the lower *θ_π_* (Table S5) in *T. truncatus* vs. *T. pueraricola* populations from the same location suggest that *T. truncatus* may have undergone stronger purifying selection despite having lower levels of genomic variation. Experimental studies suggest that rates of adaptation are linked to genome‐wide genetic diversity (Ørsted, Hoffmann, Sverrisdóttir, Nielsen, & Kristensen, 2019) but *T. truncatus* appears to have adapted despite lower diversity. It will be interesting to carry out quantitative assessments of genetically based fitness differences across populations to assess the extent to which populations from the two species are locally adapted and whether the molecular data might link to trait differentiation within and across populations (e.g., *Drosophila melanogaster* and *Carabus japonicus*; Weeks, McKechnie, & Hoffmann, 2002; Komurai, Fujisawa, Okuzaki, & Sota, 2017). The candidate genes we mentioned here are likely to provide insights into the mechanisms involved in local adaptation in spider mites, but many other significantly associated genes (Tables S12–S14) were not annotated clearly and need future exploration.

## CONFLICT OF INTEREST

None declared.

## AUTHOR CONTRIBUTIONS

X.Y.H., L.C., and J.T.S. designed the research. L.C., P.Y.J., and D.S.Z. collected materials. L.C. performed all experiments and analyses. J.T.S., P.Y.J., X.L.B., X.F.X., and A.H. took part in designing a few analyses and explaining the results. L.C., X.Y.H, and A.H. wrote the manuscript.

## Supporting information

Fig S1‐S13

Table S1‐S14

## Data Availability

All sequencing data in this study have been deposited in the NCBI database under BioProject accession PRJNA578957 (http://www.ncbi.nlm.nih.gov/bioproject/578957). Scripts used in this article are available on Github (https://github.com/Chenleinice/Codes‐for‐population‐genomic‐of‐spider‐mites).
