# Supplementary material for: Population genomic data in spider mites point to a role for local adaptation in shaping range shifts
Source: Evol Appl. 2020 Aug 27;13(10):2821–35. doi: 10.1111/eva.13086 (PMC7691463; doi:10.1111/eva.13086)
Supplement: Supplementary file 1 — Fig S1‐S13 [file EVA-13-2821-s001.pdf]

Supplemental Information Figures and Figure Legends

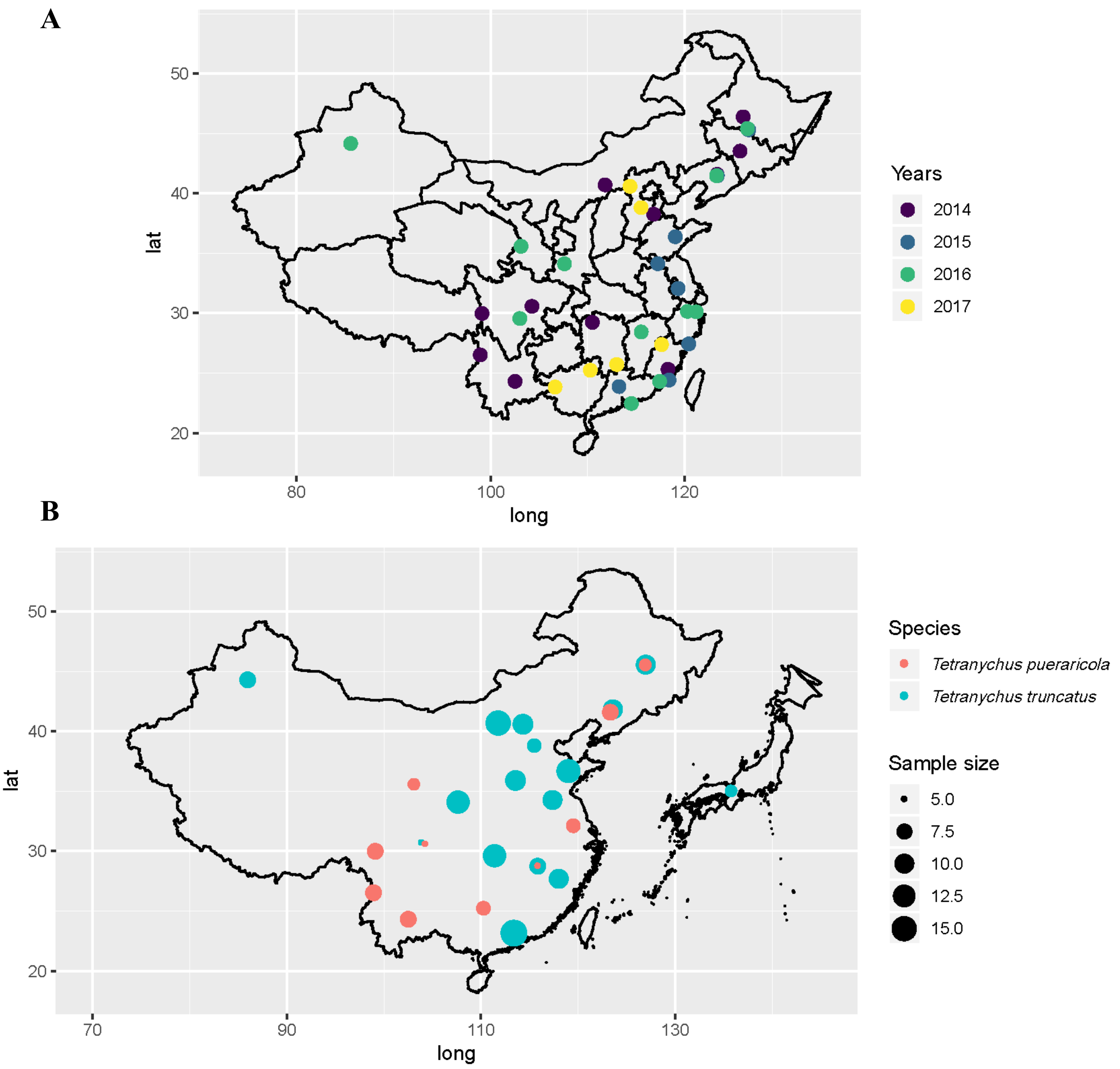

Figure S1. Maps of sampling locations. (A) Locations of 39 sites where we sampled spider mites during 2014 - 2017. (B) Locations of 173 *Tetranychus truncatus* and 67 *Tetranychus pueraricola* used for resequencing.

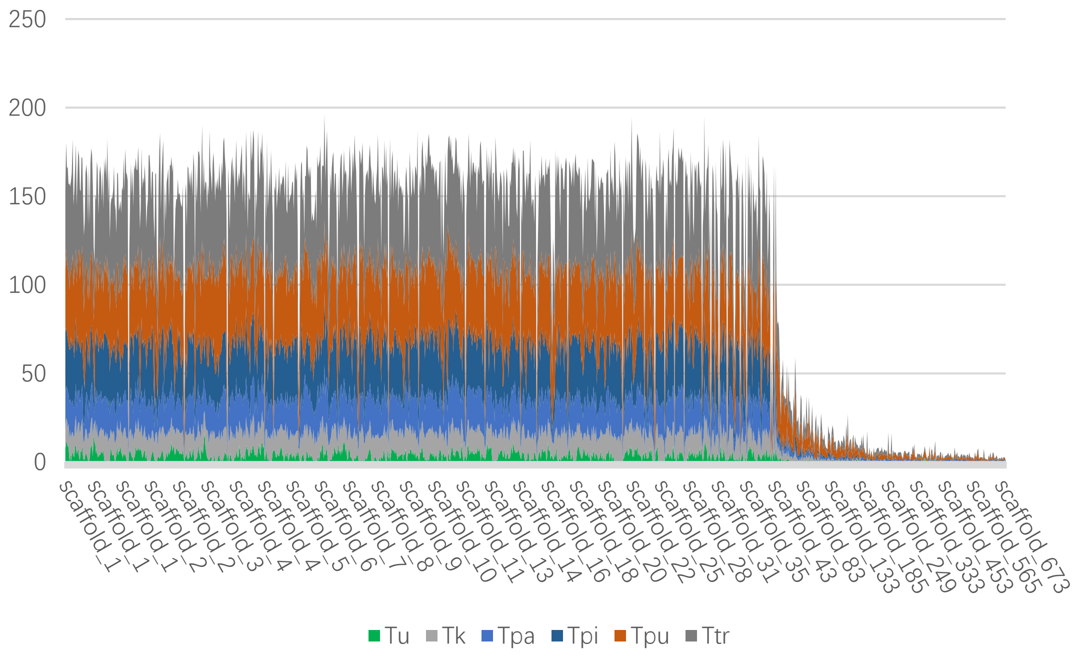

Figure S2. The distribution of SNP density of 6 spider mite species. The x axis represents 100kb size bins of scaffolds of the *T. urticae* genome. The y axis represents the density (numbers of variants per KB) of SNPs.

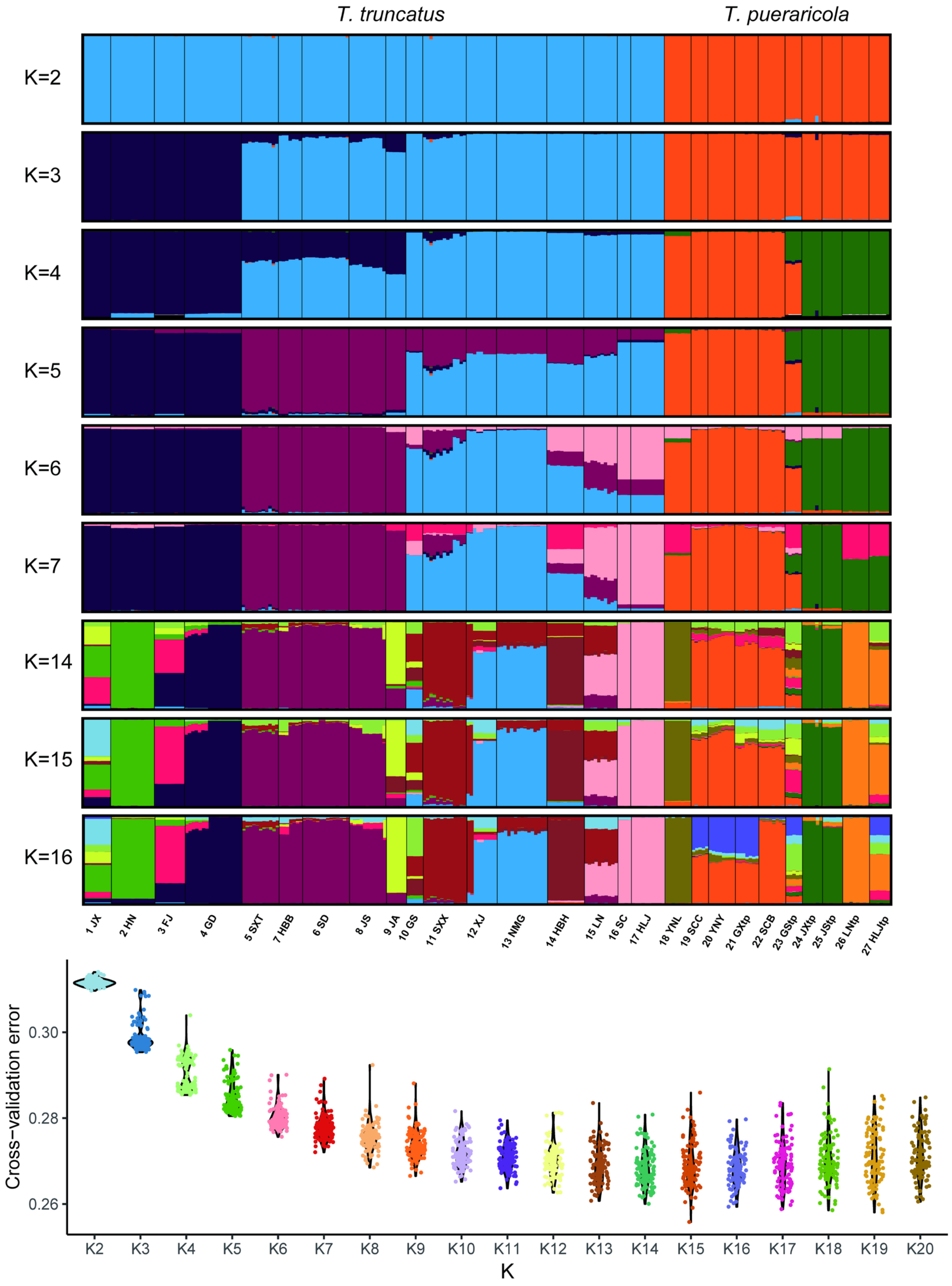

Figure S3. ADMIXTURE results and cross validation for all 27 populations of *T. truncatus* and *T. puerariicola*. In ADMIXTURE analyses we considered *K* ranging from 2 to 20 for all of the 27 populations. Here, we only displayed admixture results from *K*=2 to 7 and 14 to 16. The violin plot of cross-validation error included 100 replicates for each *K* value.

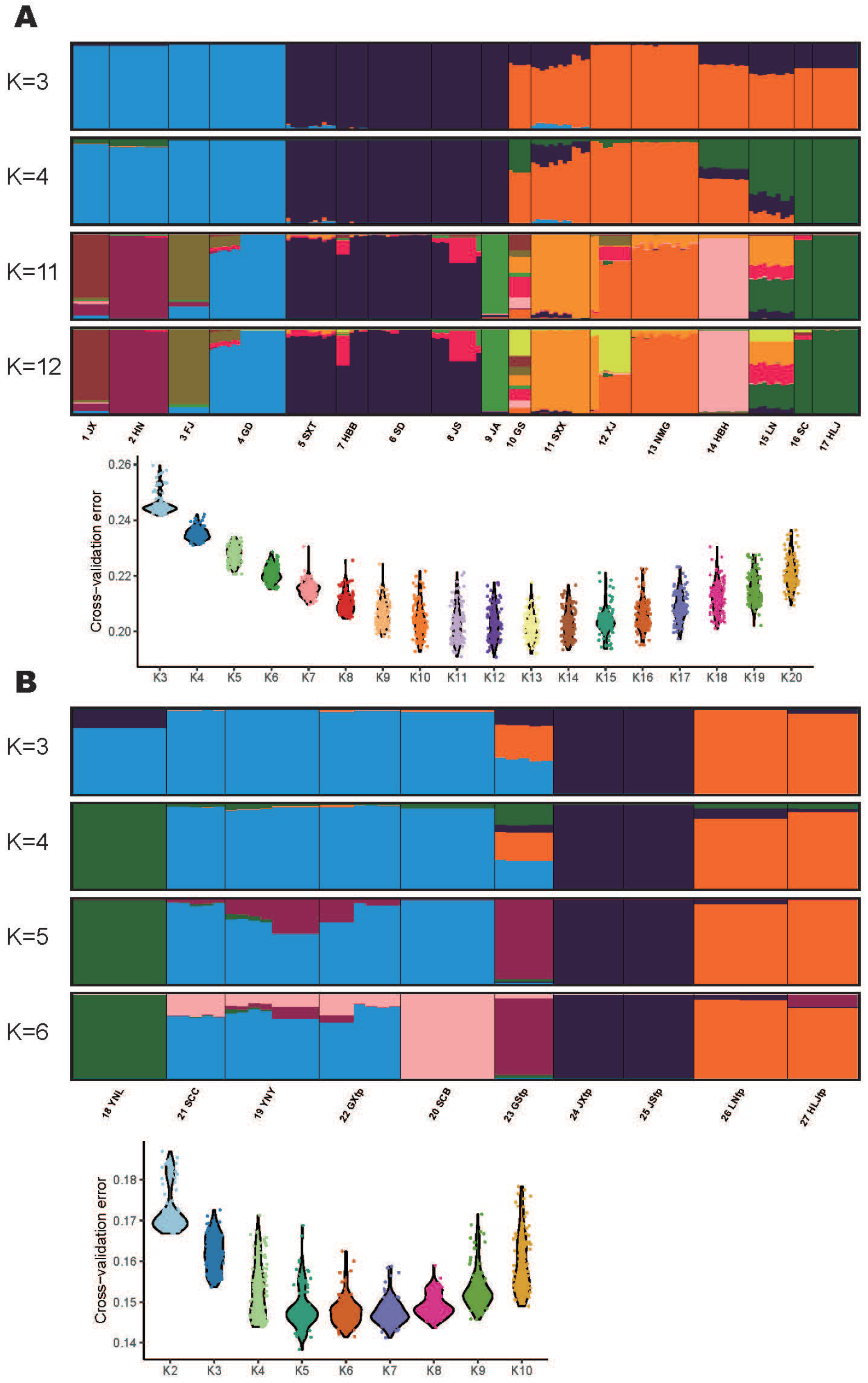

Figure S4. ADMIXTURE results and cross validation for *T. truncatus* and *T. pueraricola* respectively. (A) *T. truncatus* and (B) *T. pueraricola.*

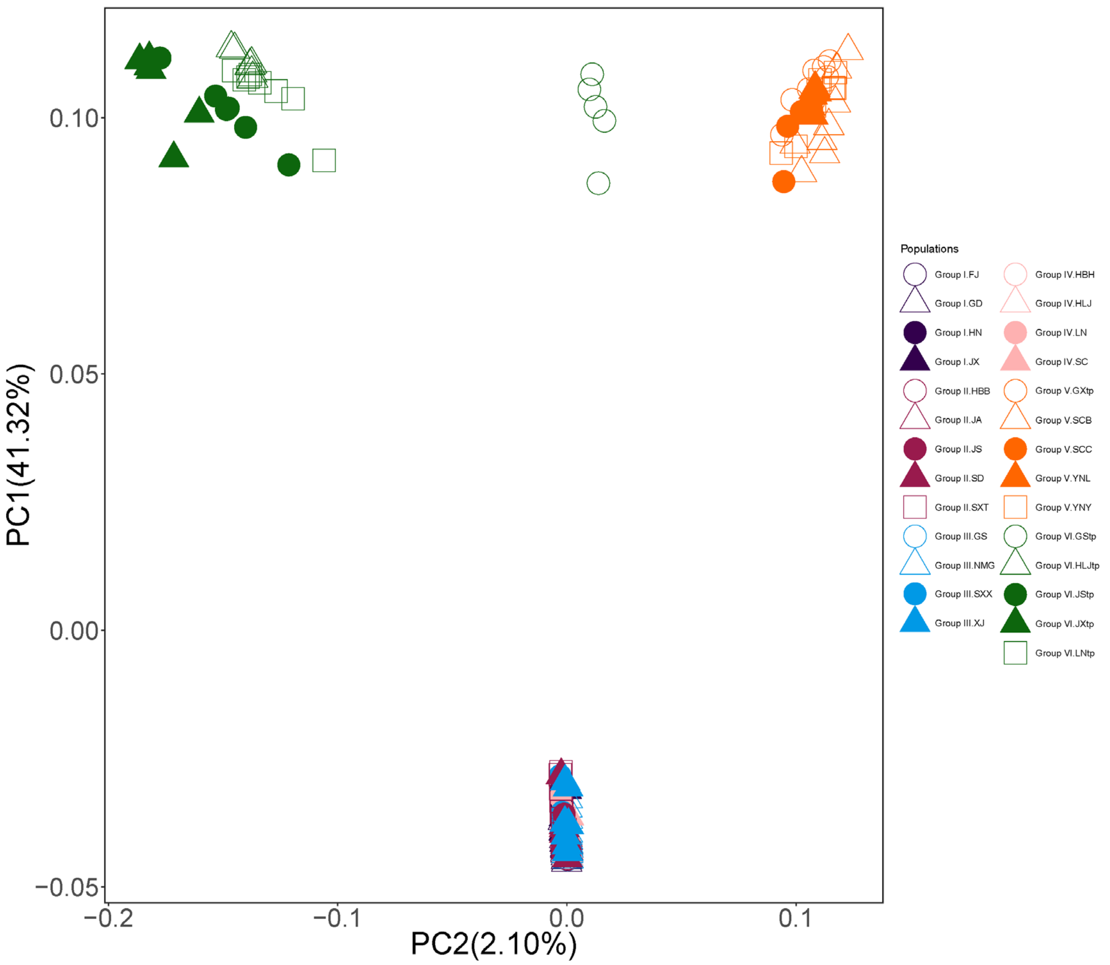

Figure S5. Principal components analysis (PCA) of *T. truncatus* and *T. puerariicola*.

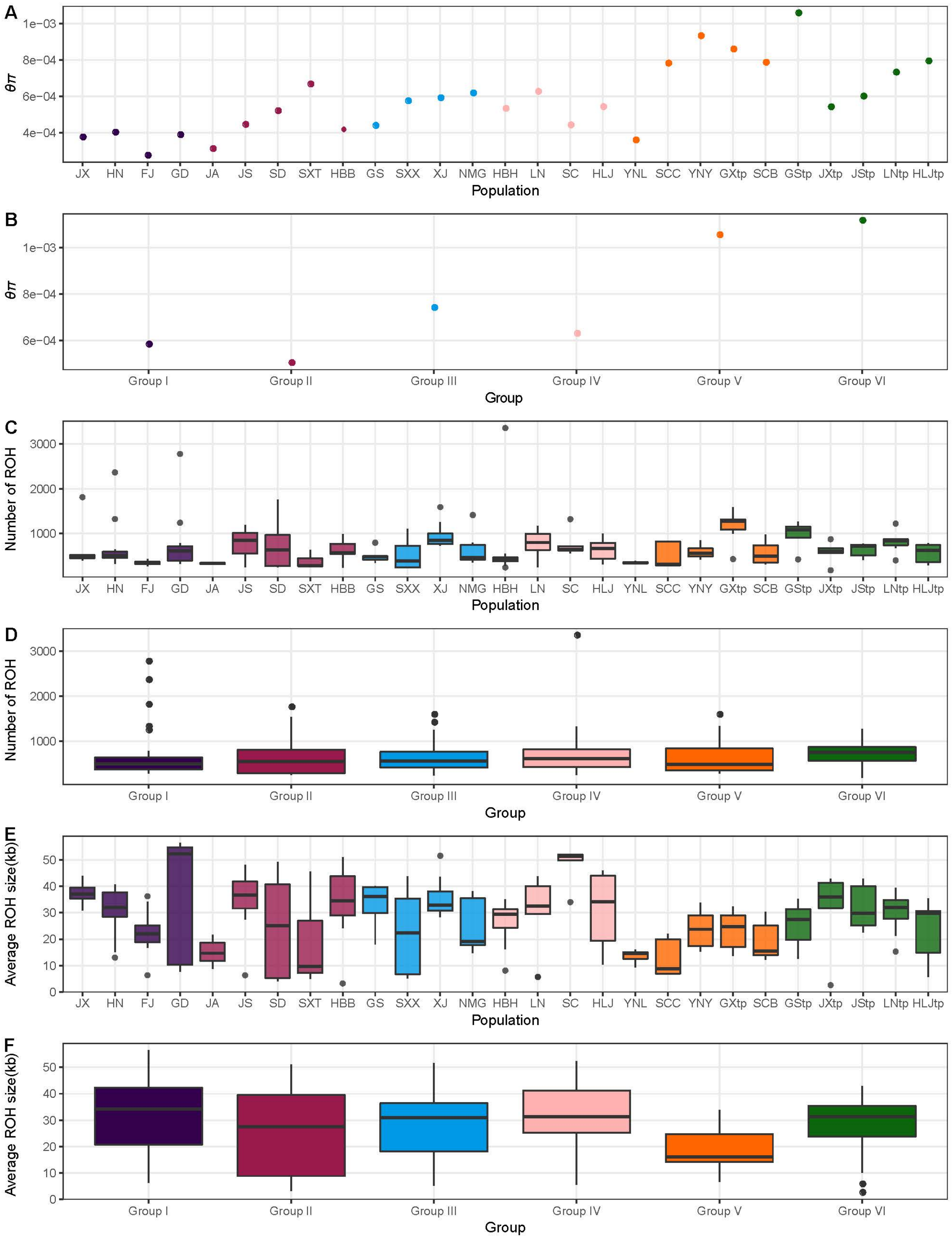

Figure S6. Summary statistics for genetic diversity and regions of homozygosity (ROH). (A) Nucleotide diversity (θ_π) of each population of *T. truncatus* and *T. pueraricola.* The colors corresponded to the different groups. (B) Nucleotide diversity (θ_π) of each group for both *T. truncatus* and *T. pueraricola.* (C) Average ROH number of all 27 populations. (D) Average ROH number in the genomes of the 6 spider mite groups. (E) Average ROH size (kb) of all 27 populations. (F) Average ROH size (kb) in the genomes of the 6 spider mite groups.

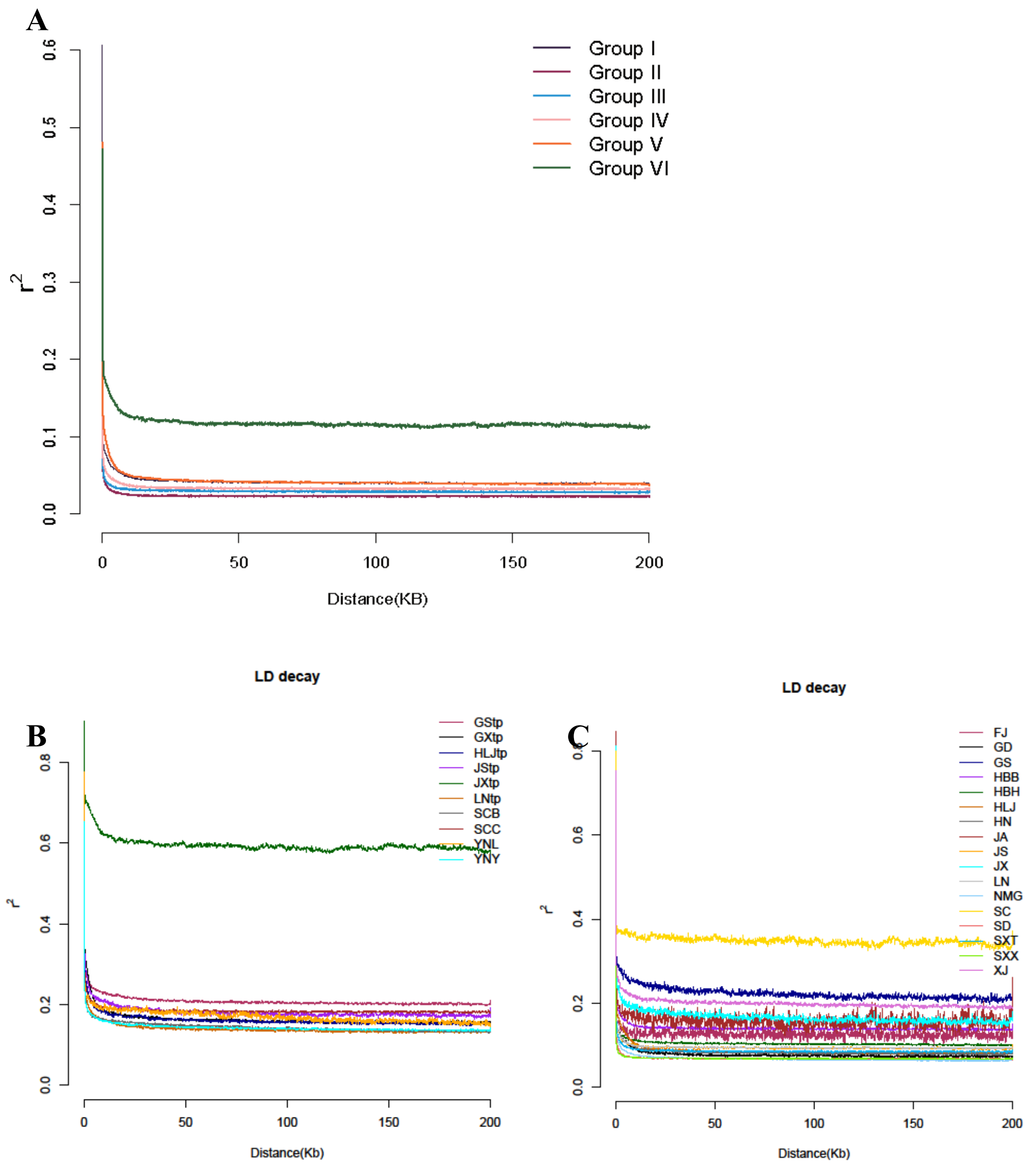

Figure S7. Decay of linkage disequilibrium (LD) in spider mite genomes. (A) Linkage disequilibrium decay in the 6 mite groups. The SC population is excluded from Group IV. (B) LD decay in all populations of *T. truncatus.* (C) LD decay in all populations of *T. pueraricola.*

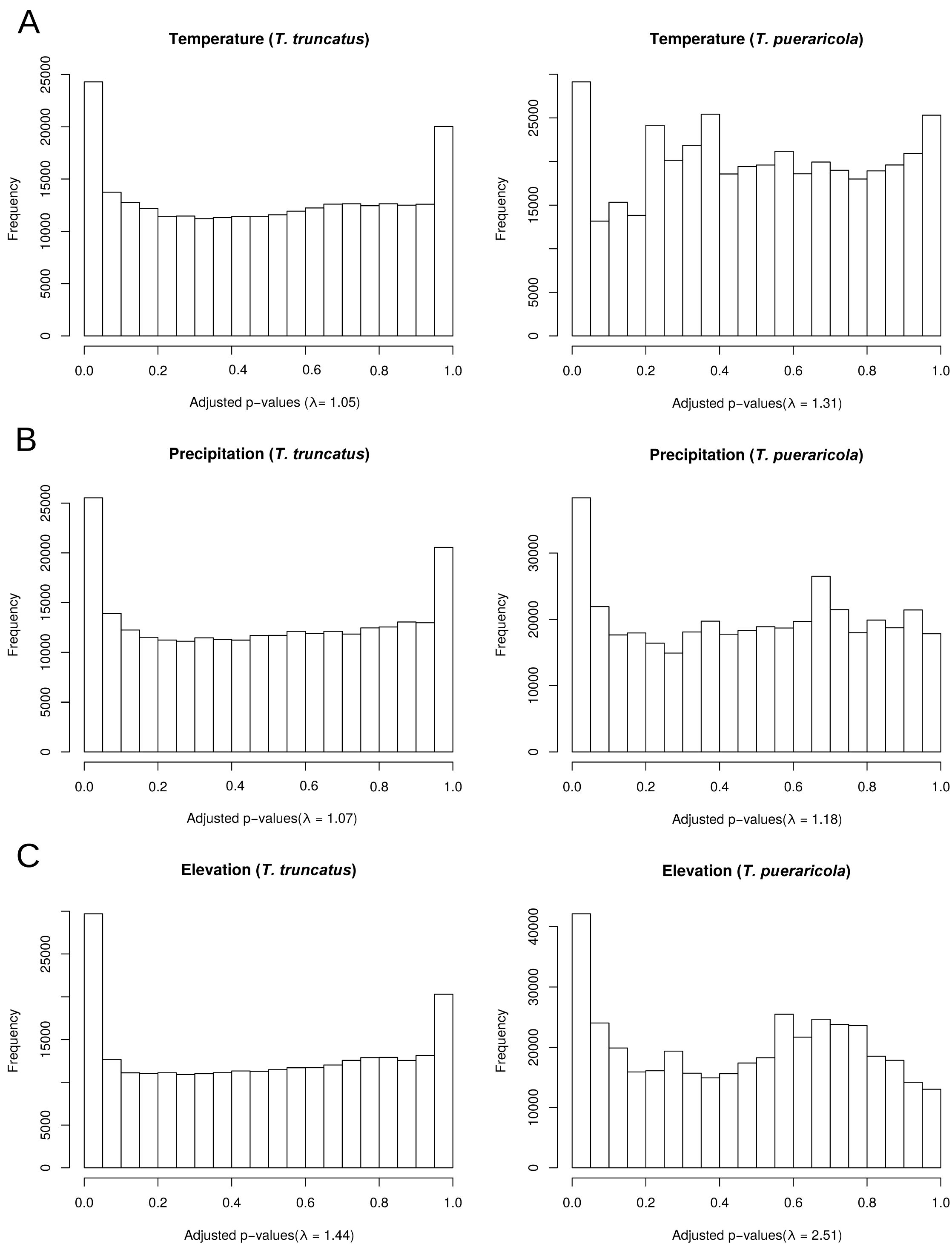

Figure S8. Histograms of adjusted p-values of climate and elevation variables. The first column includes histograms for *T. truncatus* and the second *T. pueraricola*. (A) P values of temperature variable. (B) P values of precipitation variable. (C) P values of elevation variable. Genomic inflation factors (λ) are displayed on the x axis.

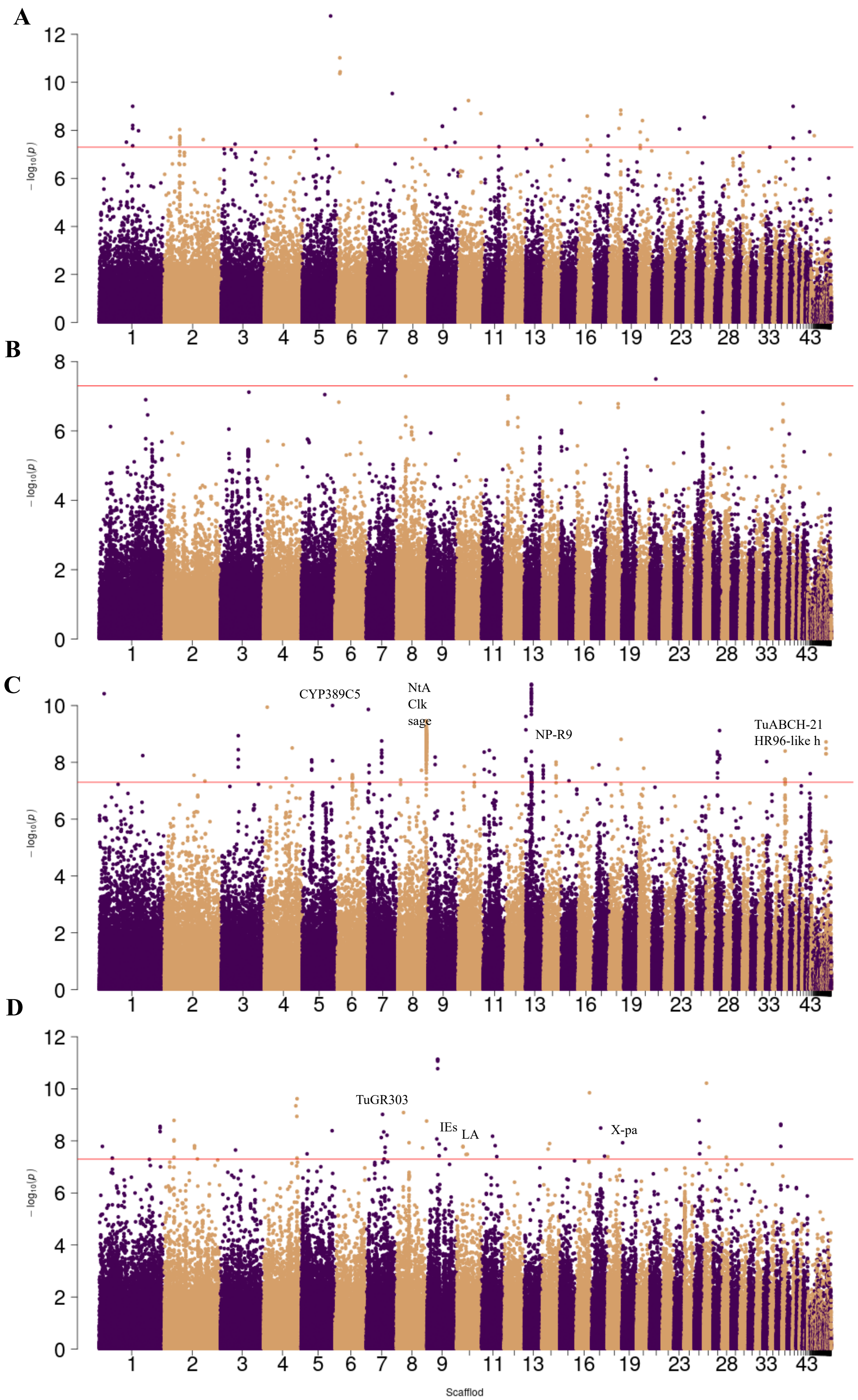

Figure S9. LFMM Analysis of the signatures of local adaptation. Manhattan plots of adjusted

p-values for SNP associations with temperature in *T. truncatus* (A) and *T. pueraricola* (B). Manhattan plots of adjusted *p*-values for SNP associations with elevation in (C) *T. truncatus* and (D) *T. pueraricola.* The genome-wide significance threshold (-log10(5e-8)) is indicated by the red horizontal line. Genes with amino acid replacement caused by significant associated SNPs (*p*-value > 5e-8) were marked. For those genes that were not named, we abbreviated them as follows: NtA (tetur08g07590, Agrin NtA), IEs (tetur09g04180, Immunoglobulin E-set), LA (tetur10g02030, laminin A) and X-pa (tetur19g00150, X-prolyl aminopeptidase).

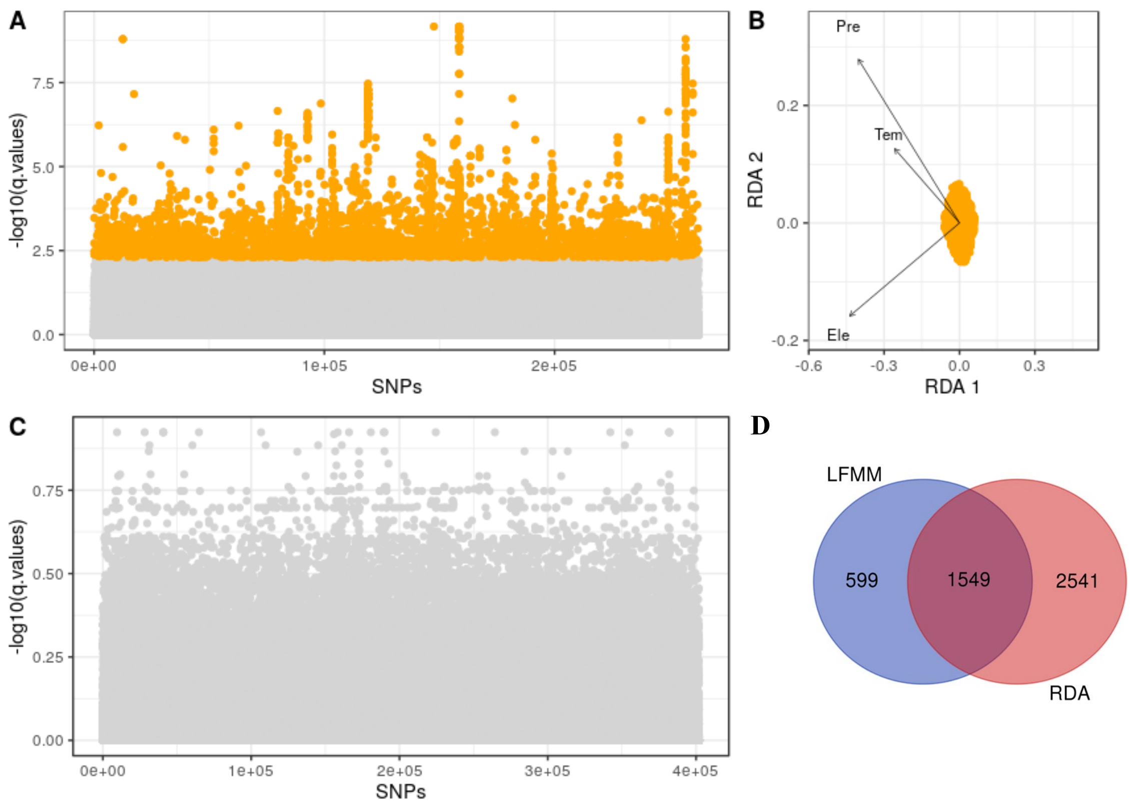

Figure S10. Redundancy analysis (RDA) to identify signatures of local adaptation. (A) Manhattan plots of adjusted *p*-values (q values) for all SNPs in *T. truncatus*. Outlier loci involved in local adaptation were marked in orange. (B) Projection of SNPs and environmental variables in the RDA performed on the adaptively enriched genetic space of *T. truncatus*. Tem, Pre and Ele represented for PCNMs for temperature, precipitation and elevation variables obtained in LFMM. The first two axes of the RDA projection represent 53% (RDA1) and 28% (RDA2) of the explained variance. (C) Manhattan plots of adjusted *p*-values (q values) for all SNPs in *T. pueraricola*. The numbers of outlier loci involved in local adaptation of *T. truncatus* and *T. pueraricola* were 4090 and 0, respectively. (D) Number of candidate/outlier SNPs involved in local adaptation identified in *T. truncatus* by the LFMM and RDA methods listed in each of the Venn diagram components.

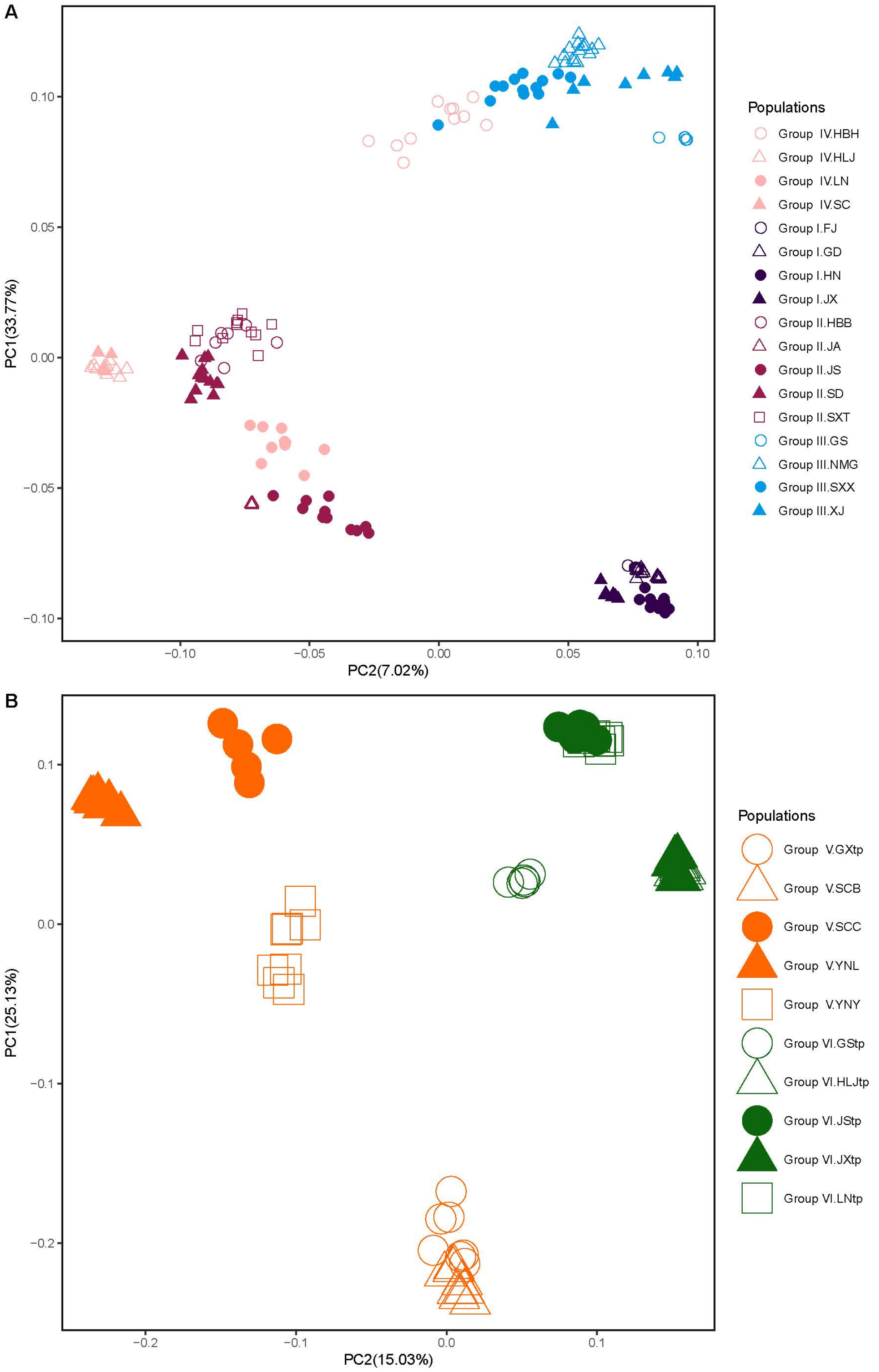

Figure S11. PCA reconstructed from the candidate SNPs associated with precipitation. (A) PCA of 4 groups of *T. truncatus*. (B) PCA of 2 groups of *T. pueraricola*. All groups and populations are represented by the combination of different colors and shapes. Extreme populations are divided according to the PC1, such as Group I and Group III of *T. truncatus*, JStp, LNtp and GXtp, SCB populations of *T. pueraricola*.

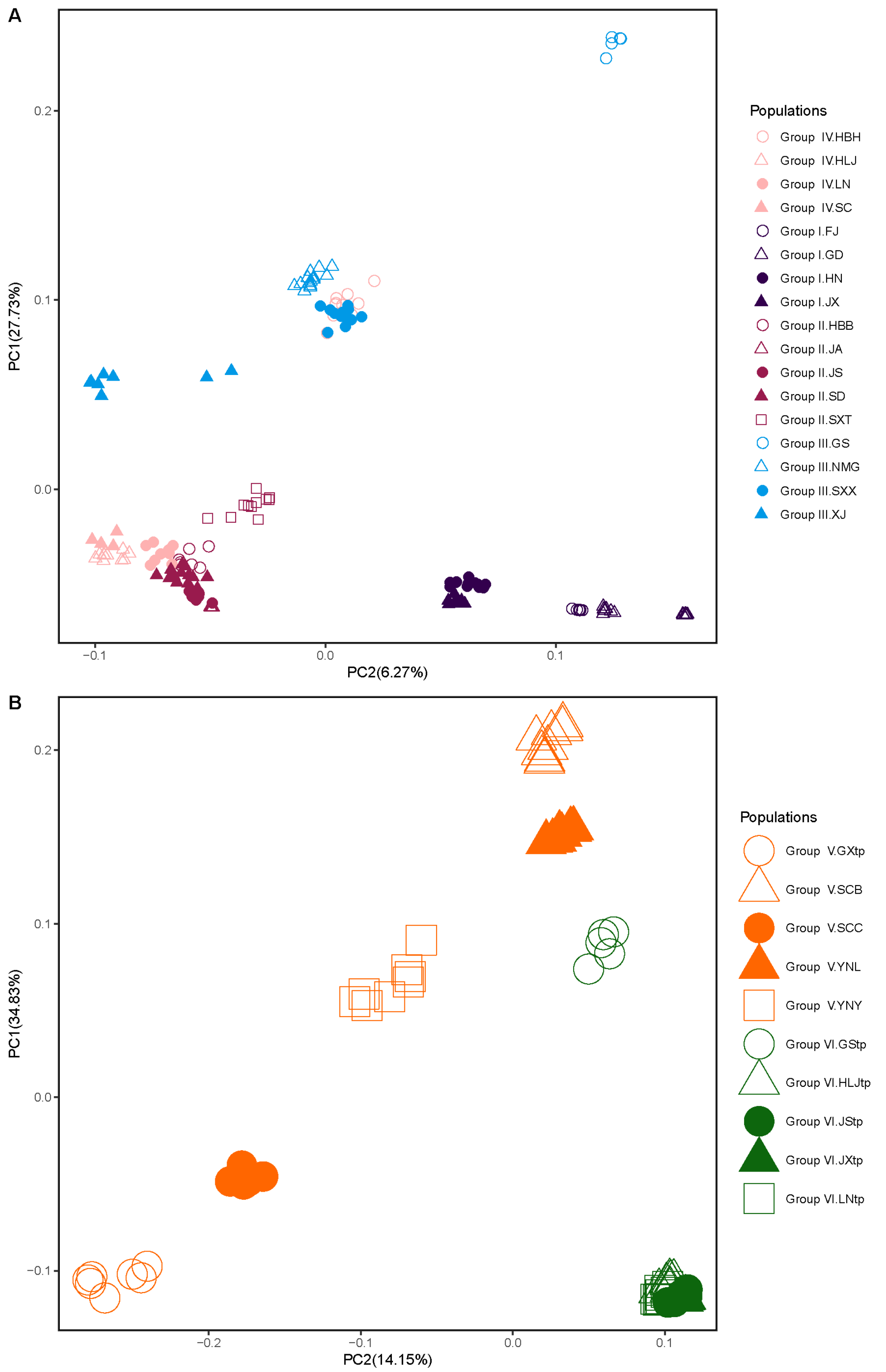

Figure S12. PCA reconstructed from the candidate SNPs associated with elevation. (A) PCA of 4 groups of *T. truncatus*. (B) PCA of 2 groups of *T. pueraricola*. Alongside PC1, populations are clustered with elevation gradient for both spider mites.

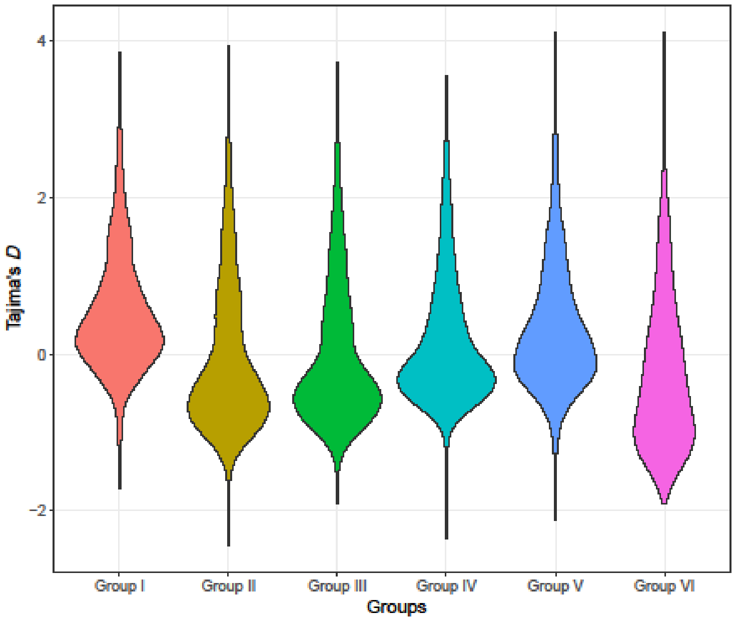

Figure S13. Summary statistics for Tajima's *D* of six groups. Tajima's *D* was calculated in non-overlapping 100-kb genomic windows. The highest Tajima's *D* of Group I indicates population contraction.
